# Modeling and Assessment of Long Afterglow Decay Curves

**DOI:** 10.1155/2014/102524

**Published:** 2014-09-14

**Authors:** Chi-Yang Tsai, Jeng-Wen Lin, Yih-Ping Huang, Yung-Chieh Huang

**Affiliations:** ^1^Graduate Institute of Dentistry, College of Oral Medicine, Taipei Medical University, Taipei 11031, Taiwan; ^2^Department of Dentistry, Taipei Medical University Hospital, Taipei 11031, Taiwan; ^3^Department of Civil Engineering, Feng Chia University, Taichung 407, Taiwan; ^4^Department of Pediatrics, Taichung Veterans General Hospital, Taichung 407, Taiwan

## Abstract

Multiple exponential equations have been successfully fitted to experimental long afterglow decay curve data for some phosphor materials by previous researchers. The calculated decay constants in such equations are used to assess the phosphorescence characteristics of an object. This study generates decay constants from experimental test data and from existing literature for comparison. It shows that the decay constants of an object may not be invariant and that they are dependent on phosphor material, temperature, irradiation intensity, sample thickness, and phosphor density for samples. In addition, the use of different numbers of exponential components in interpretation leads to different numerical results for decay constants. The relationship between the calculated decay constants and the afterglow characteristics of an object is studied and discussed in this paper. The appearance of the luminescence intensity is less correlated to the decay constants than to the time-invariant constants in an equation.

## 1. Introduction

The formation of and mechanism for phosphorescence in strontium aluminate phosphors have been thoroughly investigated, with SrAl_2_O_4_ : Eu^2+^, Dy^3+^ phosphors as one of the most studied hosts and a major host for long afterglow commercial products. The afterglow attenuates exponentially, and its intensity time decay behavior follows a first-order, second-order, or general-order kinetic behavior [1, 2]:

(1) $$\begin{array}{c} I=\frac{I_{0}}{\left(1+\gamma t\right)}\text{ first-order},\\ I=\frac{I_{0}}{{\left(1+\gamma t\right)}^{2}}\text{ second-order},\\ I=\frac{I_{0}}{{\left(1+\gamma t\right)}^{n}}\text{ general-order,} \end{array}$$

where *I* is the phosphorescence (PLUM) intensity at any time *t* after switching off the excitation illumination and *I*_0_, *n*, and *γ* are constants.

To model the measured afterglow curves, many researchers have adopted multiple (e.g., simple first-order kinetics) exponential equations as follows [3]

(2) $$\begin{array}{c} I=I_{0}+\alpha_{1}\exp\left(-\frac{t}{\tau_{1}}\right), \end{array}$$

(3) $$\begin{array}{c} I=I_{0}+\alpha_{1}\exp\left(-\frac{t}{\tau_{1}}\right)+\alpha_{2}\exp\left(-\frac{t}{\tau_{2}}\right), \end{array}$$

(4) $$\begin{array}{c} I=I_{0}+\alpha_{1}\exp\left(-\frac{t}{\tau_{1}}\right)+\alpha_{2}\exp\left(-\frac{t}{\tau_{2}}\right)+\alpha_{3}^{}\exp\left(-\frac{t}{\tau_{3}}\right), \end{array}$$

where *I*, *I*_0_, and *t* are as defined in (1), *α*_*i*_ are time-invariant constants, and *τ*_*i*_ are the decay constants (also called decay times) of the exponential components.

The decay constants are a means to determine the decay rate of the rapid, medium, and slow exponential decay components. The single (2), double (3), or triple (4) exponential equations have been widely used in recent research that fitted the experimental decay curves and may correspond to one, two, or three trap centers in the assumed model. Tsai et al. [4] have dealt with the mathematical modeling problems and have suggested that the slope change of the curve profile is determined by *τ*_*i*_ in the equation and that it has nothing to do with the magnitude of luminous intensity; they also examined the effect of the offset term (*I*_0_) in an equation that had been fitted to experimental data for applications and claimed that this term should not occur in a good experimental environment [5].

By using the triple exponential equation (4), Wu et al. [6] discovered that increasing the environmental temperature decreases the value of *τ*_3_ (the largest decay constant that dominates the duration of the long afterglow). Kubo et al. [7] adopted the double exponential equations (i.e., (3)) in their analysis and claimed that the PLUM intensity of the slower decay component (larger one) decreased with temperature in the range from 305 to 458 K. Wu et al. [8] suggested that a general-order kinetics model provided the best fit for the data in their test as well as accommodating retrapping in the thermoluminescence and decay processes. He et al. [9] studied the charging process efficiency and assumed for simplicity that the traps have a single depth and hence that it was reasonable to use a single exponential equation (2) in the curve fitting process. Moreover, they pointed out that the PLUM was more intense as one pumped more irradiation energy after the fluorescence was first saturated.

Other studies considered the calculated decay constants as indicative of trap depths to interpret the physical afterglow behaviors. Zhu et al. [10] calculated the constants and indicated that the decay times for phosphors were prolonged by encapsulation at room temperature. Xie et al. [11] showed that the decay characteristics reflected the fact that phosphors with different structures possessed different afterglow times. Chang et al. [12] concluded that the larger the value of the decay constant was, the slower the decay speed and the better the afterglow properties were. Pedroza-Montero et al. [13] found that the PLUM of SrAl_2_O_4_ : Eu^2+^, Dy^3+^ phosphors was characterized by at least three temporal processes around 0–25 s, 25–200 s, and 200–650 s. The most intense PLUM came from the fast decay parts (0–25 s and 25–200 s). The slower part (200–650 s) was the most enhanced for higher doses. Arellano-Tánori et al. [14] reviewed the real PLUM decay process and reported that it is complicated and that an exponential-type decay model was an oversimplification. The PLUM exhibited an intensity time decay behavior composed of three simple exponential terms with decay constants of 56 s, 180 s, and 1230 s.

Han et al. [15] stressed that mere fitting of data was not physical evidence for the existence of only two trapping levels of different energies. There may be more than two trapping levels as suggested by many researchers. Nevertheless, they could always be averaged into two types of trapping levels, that is, shallow and deep. Meléndrez et al. [16] suggested that it is reasonable to assume that the PLUM intensity profiles depend on irradiation temperature and irradiation dose exposure.

Based on the aforementioned arguments, four points are raised as follows.

1. By using single, double, or triple exponential equations in curve fitting for a case, different decay constants may be derived for the calculated parameters.
2. Is the lower-order component of the decay constants the most enhanced for higher doses? Does the larger value of this lower component correspond to better afterglow properties?
3. If the PLUM intensity profiles depend on the irradiation dose exposure, how do the related decay constants respond?
4. What are the major influencing factors on the three types of parameters in the above equations, that is, *I*_0_, *α*_*i*_, and *τ*_*i*_?

This study intends to answer these four points. Several forms of the decay curves have been collected, including those from existing articles, luminescence standards, and experimental tests conducted in the lab. To model and compute the decay constants of the long afterglow decay curve of the material studied, the exponential equations (2)–(4) were fitted to experimental data and the data were generated from referenced articles using a modified least squares method. We focus on the study of physical glow behaviors, or the so-called photoluminescence, not luminescence arising from chemical or other forms of reactions.

## 2. Experimental Tests

This study uses PET (polyethylene terephthalate) resin as a matrix combined with strontium aluminate phosphors to form afterglow luminous thick films, with the ultimate aim of increasing its luminous intensity. Besides the phosphor material, the resin material, forming process, and physical properties are vitally important in the afterglow end product performance. The various specimens investigated in this study are manufactured with the same phosphor density but different thicknesses. The phosphor particle size is 0.1 mm in diameter, and the phosphor density in the mixture is 1 : 1 (PET versus phosphors). We use a mesh print technique to create the patches, printing layer by layer on top of a substrate with each layer being about 0.1 mm thick. In other words, to obtain a 0.6 mm thick patch, it was necessary to make six runs of the print process.

The patch thickness and exposure dose are two major factors considered in this study. DIN 67510 and JIS 9107-2008 [20] luminescence standards were adopted.  Table 1 lists four test conditions used in this study for comparison. The room temperature in the experimental tests was 25°C.

### 2.1. Materials

In this study, the investigated specimens were made by using SrAl_2_O_4_ : Eu^2+^, Dy^3+^ phosphor powder mixed with 60% PET resin in weight, which was printed on top of the PET plastic white substrate to form a plastic patch with thicknesses between 0.4 mm and 0.6 mm.

### 2.2. Photoluminescence Experimental Test

An oven was designed for this study, inside which there are four 6500 K, 100 W adjustable intensity Xenon lamps, attached to the top and sidewalls to excite afterglow specimens. The lamps could be switched on independently to meet the required luminescence intensity. A transparent glass door was installed behind the metal door in order that the metal door might be opened for monitoring the luminescent behavior of specimens without disturbing the interior temperature of the oven.

A computer-controlled luminance meter (Konica Minolta LS-100) was used to measure the luminous intensity of the afterglow specimens of this study. In order to keep the same irradiation conditions among different tests before irradiating samples, samples were heated to 70°C for 6 hours to remove any acquired and residual thermoluminescence prior to cooling to the desired experimental temperature.

## 3. Slow Decay Component

As in (4), the terms on the right hand side of the equations with subscripts 1, 2, and 3 are assigned as fast, medium, and slow components. In other words, they are the terms representing the major influences for different periods of afterglow luminescence duration. At large *t*, the dominant term is the one corresponding to the slowest component. This may be extracted as

(5) $$\begin{array}{c} I_{L}=\alpha_{L}\exp\left(-\frac{t}{\tau_{L}}\right), \end{array}$$

in which *I*_*L*_ is the long term intensity at time *t*, *α*_*L*_ is the last time-invariant constant corresponding to the slowest decay component (i.e., *α*_2_ in (3) or *α*_3_ in (4)), and *τ*_*L*_ is the corresponding decay constant.

**Figure 1:**
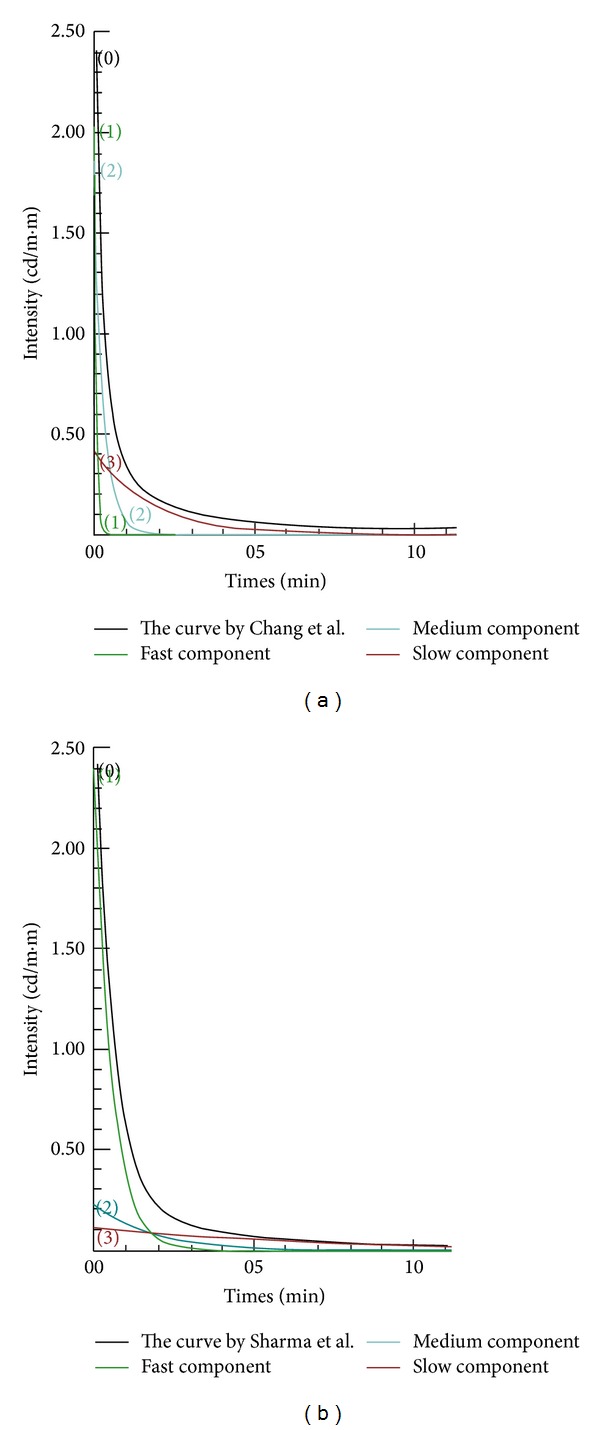
The figures of fast, medium, and slow component in an equation. Data cited from [12, 17].

In the afterglow luminescence, the slow component is responsible for the long persistent behavior [19]; it shows that the initial intensity for this term is *α*_*L*_, and the decay rate of the luminous intensity is proportional to *τ*_*L*_; that is, larger values for *α*_*L*_ and *τ*_*L*_ would result in a higher intensity for (5).  Figure 1 displays two figures cited from two articles [12, 17]. The right hand side figure shows that the three components (fast, medium, and slow) of a curve intersect at about 1.9 min, and the slow decay component, as shown in (5), is the major contributor to the luminous light. The left hand side figure shows that the slow component becomes dominant after 1 min compared to the other two components. This is the major reason researchers focus their studies on the slow component term in an equation when dealing with afterglow luminescent related topics.

## 4. Numerical Simulations

Many researchers [2, 3, 6–16] have assessed the phosphorescent characteristics according to the decay constants calculated from the use of curve fitting techniques. Decay curves were fitted to the sum of a few exponential components, each with its own decay constant. Several computer programs were written for this study to calculate the related constants from the experimental data and also other data from referenced articles.

The following equation is a matrix expression for a linear relationship between a set of data and a set of parameters:

(6) $$\begin{array}{c} {\left[A\right]}_{n\times m}{\left\{X\right\}}_{m\times 1}={\left\{B\right\}}_{n\times 1}, \end{array}$$

in which *A* is a coefficient matrix, *n* is the number of components in an equation, *m* is the number of data points, *X* is the parameters of the components, and *B* is the known data.

Equations (2) to (4) are nonlinear equations. The three types of parameters to be calculated include *α*_*i*_, *τ*_*i*_, and *I*_0_. The process and procedures used in this study are described as follows:

(1) some values were assigned to *τ*_*i*_ with the restriction *τ*_*i*_ < *τ*_*i*+1_ to use any one of (2)–(4);
(2) the processed exponential equation could be transformed to a linear equation as in (6); *α*_*i*_ and *I*_0_ were then calculated using the least squares method to simulate a set of experimental data;
(3) any predefined *τ*_*i*_ could generate an associated deviation (*D*) by using (7) to represent the difference between the computed values and the used data set:
  (7) $$\begin{array}{c} D=\sqrt{\frac{\sum_{i=1}^{q}{\left[y_{i}-f(x_{i})\right]}^{2}}{q},} \end{array}$$
  where the deviation, *D*, is defined as the root mean square offset between the experimental data *y*_*i*_ and the computed value *f*(*x*_*i*_) through the use of one of (2)–(4);
(4) as the *τ*_*i*_ sampled a wide range of values, the minimum deviation case was revealed. However, in addition to the minimum deviation concerned, a minimum ∑*τ*_*i*_ total was employed to filter out the unfit equations.

There are two different schemes proposed in this study to execute the least squares method. They are described in the following two subsections.

### 4.1. Exhaustive Search

This search process was to set fixed ranges for *τ*_*i*_ values with a constant increment and the restriction that *τ*_*i*_ < *τ*_*i*+1_. For example, the search range for *τ*_1_ is from 1 to 1.0 with 0.001 increment, that is, 1000 elements in the range. For *τ*_2_, it is 1.5 to 3.0 with 0.001 as increment, and for *τ*_3_, it is 4.0 to 10.0 with 0.002 increment. The relative ordering *τ*_1_ < *τ*_2_ < *τ*_3_ was retained in this process.

This straightforward method searches through a predefined range for *τ*_*i*_ and records the calculated *D* (deviation), *I*_0_, and *α*_*i*_. Then, it is possible to choose one set of *α*_*i*_ and *τ*_*i*_ that fit with the minimum *D* and some predefined criteria.

### 4.2. Branch Search

The exhaustive search method required searching with a wide fixed range and inevitably was CPU-intensive. To be more specific in setting appropriate search ranges, a branch search method was proposed. This study used (2) to process the single exponential equation first and the obtained value *τ*_1_* was used to estimate the search ranges for the double exponential. The obtained values *τ*_1_** and *τ*_2_** were then used to set the ranges for the triple exponential. The three process steps may be described as follows.

(*1) Single Exponential Equation.* Consider

(8) $$\begin{array}{c} I=I_{0}+\alpha_{1}\exp\left(-\frac{t}{\tau_{1}}\right). \end{array}$$

Two values are assigned as boundaries, *τ*_min⁡_ and *τ*_max⁡_, and an increment is assigned to process the problem:

(9) $$\begin{array}{c} \tau_{\min}<\;\tau_{1}<\tau_{\max}. \end{array}$$

The least squares method is first used to find *τ*_1_* with the minimum deviation among all. In order to achieve better results, the searching ranges were narrowed down as *τ*_1_* ± increment and the search was repeated until the increment was small enough.

(*2) Double Exponential Equation.* Consider

(10) $$\begin{array}{c} I=I_{0}+\alpha_{1}\exp\left(-\frac{t}{\tau_{1}}\right)+\alpha_{2}\exp\left(-\frac{t}{\tau_{2}}\right). \end{array}$$

In this step, the calculated *τ*_1_*, *τ*_min⁡_, and *τ*_max⁡_ are used to set the ranges as

(11) $$\begin{array}{c} \tau_{\min}<\tau_{1}<\tau_{1}^{*},\;\;\tau_{1}^{*}<\tau_{2}<\tau_{\max}. \end{array}$$

The least squares method is used to find a pair of values *τ*_1_** and *τ*_2_** with the minimum deviation. The parameters can be fine-tuned by using *τ*_1_** ± increment and *τ*_2_** ± increment as two separate ranges for further convergence processes.

(*3) Triple Exponential Equation.* Consider

(12) $$\begin{array}{c} I=I_{0}+\alpha_{1}\exp\left(-\frac{t}{\tau_{1}}\right)+\alpha_{2}\exp\left(-\frac{t}{\tau_{2}}\right)+\alpha_{3}\exp\left(-\frac{t}{\tau_{3}}\right). \end{array}$$

Again, the calculated *τ*_1_**, *τ*_2_**, *τ*_min⁡_, and *τ*_max⁡_ are provided to set the following ranges as:

(13) $$\begin{array}{c} \tau_{\min}<\tau_{1}<\tau_{1}^{**},\;\;\tau_{1}^{**}<\tau_{2}<\tau_{2}^{**},\\ \tau_{2}^{**}<\tau_{3}<\tau_{\max}. \end{array}$$

Once again, we use the least squares method to find a set of *τ*_1_***, *τ*_2_***, and *τ*_3_*** with the minimum deviation. Further processes continued in the same way as in the previous double exponential case.

In general, the branch search method may reduce search CPU time, as it spends about 1 min of CPU time to search for an optimal single, double, and triple exponential equations simultaneously for all the cases used in this study. However, it may fail for a few cases, since if the single exponential solution *τ*_1_* is not appropriate, then it may also fail to converge for the double and triple cases.

## 5. Interpretation of Data 

Data from various resources were derived via different means described as follows.

1. Data from existing articles: previous studies have provided a set of well-documented constants in a specified multiple exponential form. This study uses these constants to regenerate point data for the decay curve, and then the decay constants of the single, double, and triple multiple exponential equations were calculated separately.
2. Data referred to the DIN standard: those data represent some points on the associated decay curves. This study used the data to compute the associated decay constants for the double exponential equation forms as in (3).
3. Data generated from the experimental tests in this study: in order to study the effect of the irradiation duration as well as the dose exposure on the PLUM intensity profile of the phosphorescent material, we provided data from several experimental tests for use.

### 5.1. Data from Existing Articles

Table 2 contains two different data types in the original sources: one is the result from triple exponential equations and the other is the result from the double exponential equations. Table 2 shows the deviations resulting from all three types of exponential equations and indicates that the single exponential equations always resulted in larger deviations than the double or triple ones. The simulation deviations induced in the triple exponential equations were the smallest among them, which means that the use of triple exponential equations in interpretation may be the best model among the three. Table 2 indicates that the *τ*_2_ in the double exponential equation were closely associated with *τ*_3_ in the triple exponential equation. Similar relationships were found in *α*_2_ and *α*_3_ constants. The averages of the three decay constants of the triple exponential equations were 18.45, 91.34, and 360 s, which fall within the range suggested by Pedroza-Montero et al. [13].

**Figure 2:**
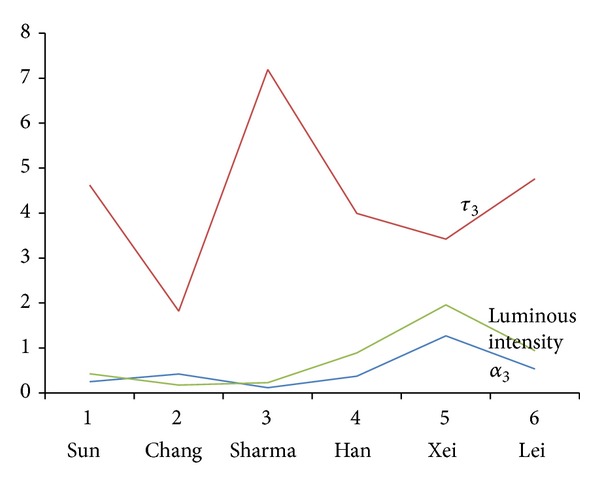
Relationships between the luminous intensity (measured at 2 min), *α*_3_, and *τ*_3_ for the data listed in Table 2.

Figure 2 illustrates the relationship among the luminous intensity, *τ*_3_, and *α*_3_ (Table 2). This figure indicates that the value *α*_3_, rather than *τ*_3_, is most strongly correlated with the associated luminous intensity. It is noted that Chang et al. [12] used Sr_3_Al_2_O_6_ : Eu^3+^, Dy^3+^ that could reflect different decay characteristics. That is to say they did not use the same material compared to others. Hence, it is reasonable to ignore this point of Figure 2. The remaining data show consistent behavior and a high correlation between the luminous intensity and *α*_3_ but it is hard to find any relationships between the luminous intensity and *τ*_3_.

### 5.2. Data from the DIN Standard

**Figure 3:**
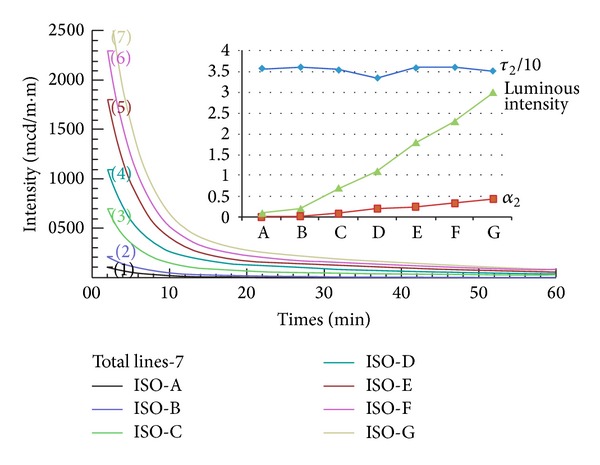
Afterglow profiles of the DIN luminous standard where the luminous intensity is measured at 2 min.

Figure 3 shows the luminous profiles as provided by the DIN standard that grades from A to G for industry usage.  Table 3 shows the calculated constants corresponding to the profiles in the figure. Again, the use of single exponential equation in interpretation results in the largest deviations and the smallest *τ*_1_ value. Also, it indicates that the grade with the largest luminous profile (G) does not necessarily have the largest decay constant (*τ*_3_).

This finding seems inconsistent with the argument of Chang et al. [12], who stated that the larger the value of decay time was, the better the afterglow properties were. On the contrary, this study indicates that larger *α*_3_ values are associated with the better afterglow properties. This statement is also true for those cases in Table 2. The upper right hand side of Figure 3 displays the curves for luminous intensity, *α*_3_, and *τ*_3_, which provides evidence that *α*_3_ is more directly correlated with the luminous intensity than *τ*_3_.

### 5.3. Data from Experimental Tests

Table 4 lists the results of the experimental tests conducted in this study. There are two different thicknesses for the patches, that is, 0.4 mm and 0.6 mm.  Table 1 shows the four different luminous intensities used to irradiate the specimens. The irradiation conditions follow the DIN and JIS luminance standards.

**Figure 4:**
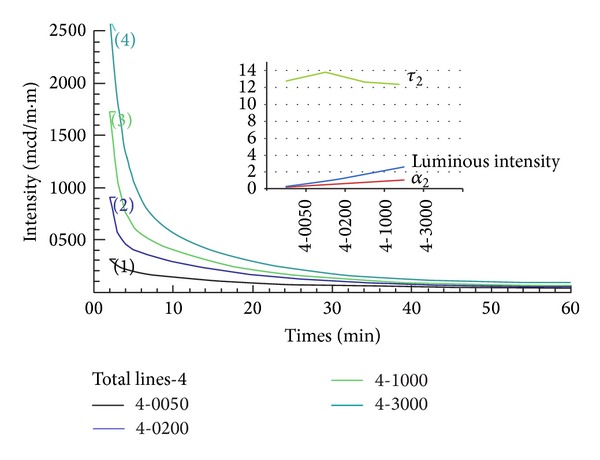
Afterglow profiles of the 0.4 mm thickness patch where the luminous intensity is measured at 2 min.

**Figure 5:**
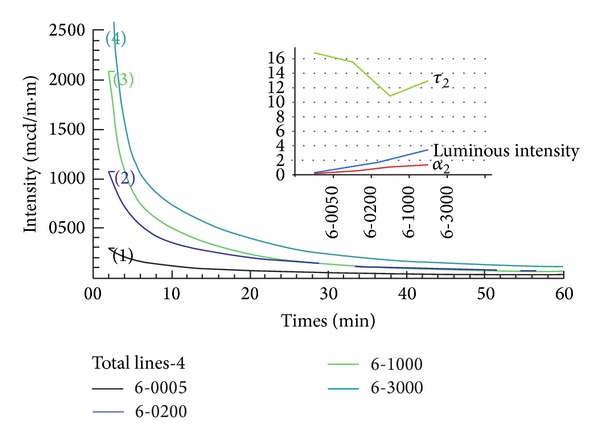
Afterglow profiles of the 0.6 mm thickness patch where the luminous intensity is measured at 2 min.

The corresponding luminescence decay curves are shown in Figures 4 and 5 for 0.4-mm and 0.6 mm thickness patches, respectively. It is obvious that the higher irradiation intensity and thicker specimens achieved higher afterglow luminous intensity after input illumination has ceased. As expected, the thicker the patch was, the better afterglow luminous behavior was. For the same patch, better luminous behavior was accompanied by higher *α*_3_ constants as depicted at the upper right hand side of Figures 4 and 5. On the other hand, the *τ*_2_ curve behaves differently in these two figures.  Figure 4 shows that *τ*_2_ remain almost constant under different irradiation intensity. However, in contradiction to this, Figure 5 indicates that *τ*_2_ decreases with increasing irradiation intensity in a test.

## 6. Conclusions

For an afterglow decay profile, it is important to adopt appropriate multiple single exponential equations in the associated numerical simulation. Single, double, and triple exponential equations have been applied in turn to approximate a set of data. Then, the one with the minimum deviation and ∑*τ*_*i*_ total was chosen. To solve the nonlinear simultaneous equations involved, the exhaustive search and brand search methods have been used for all the cases mentioned. Different decay constants were obtained for the calculated parameters depending on whether single, double, or triple exponentials were used to fit the curves for each case. With more exponential terms (say *n* terms) used in an equation to simulate a set of data, smaller *α*_*n*_ and larger *τ*_*n*_ values are found in the results.

With the commonly used strontium aluminate phosphors, the data from previous and present experimental work indicated that the last *α*_*i*_, rather than the last *τ*_*i*_, in an exponential equation is most strongly correlated with the afterglow characteristics of an object. The last *α*_*i*_ value is a good index reflecting the amount of irradiation dose exposure and the quality of afterglow properties (Figures 3, 4, and 5). However, the consistent behaviors between luminous intensity and the *α*_3_ and *τ*_3_ values in the same afterglow phosphors do not exist among different phosphors. These findings are contradictory to other studies ([12, 14, 15]), which have correlated luminous intensity with only the value of *τ*_3_. Thus, we conclude that it is important to carry out further studies of this nature in order to verify this finding. As shown in Figure 2, our approach may not be appropriate to cross link parameters between different systems; this needs further detailed study. Moreover, other afterglow characteristics such as the temperature effect, or different phosphor materials, also need to be studied.

## Figures and Tables

**Table 1:** The luminous intensity and exposure duration used in this study.

Regulation code	Luminous intensity (lx)	Duration (min)
DIN 67510	1000 50	5 15
JIS Z 9100	200	20
This study	3000	5

**Table 2:** Fitting parameters of the decay curves by using the single, double, and triple exponential equations. -1, -2, and -3 indicate the use of single, double, and triple exponential equations in interpretation, respectively.

Sources	Deviation %	*I*_0_	α_1_	α_2_	α_3_	τ_1_ (min)	τ_2_ (min)	τ_3_ (min)
Sun-1 [18]	0.1008	0.3034	3.6530			0.2333		
Sun-2	0.0020	0.2205	3.5206	**0.2985**		0.3176	**4.1248**	
Sun-3	(given)	0.2200	2.8790	0.6890	**0.2520**	0.1280	0.7227	**4.6237**

Chang-1 [12]	0.0392	0.0687	4.2653			0.3657		
Chang-2	0.0019	0.0316	3.8760	**0.4269**		0.2371	**1.8048**	
Chang-3	(given)	0.0315	2.0270	1.8547	**0.4212**	0.0567	0.2900	**1.8200**

Sharma-1 [17]	0.0548	0.0	2.8928			0.6900		
Sharma-2	0.0036	0.0	2.7457	**0.2431**		0.5500	**4.3567**	
Sharma-3	(given)	0.0	2.6416	0.2344	**0.1153**	0.5328	1.8554	**7.1918**

HAN-1 [15]	0.0664	0.0684	8.8483			0.88002		
HAN-2	(given)	0.0082	7.9885	**0.6772**		0.7789	**3.2925**	
HAN-3	0.00085	0.0071	7.9132	0.3798	**0.3733**	0.7250	2.2800	**3.9900**

Xei-1 [11]	0.1350	0.0	6.7849			0.898		
Xei-2	(given)	0.0	4.4678	**2.4138**		0.2523	**2.6139**	
Xei-3	0.0107	0.0	4.3009	1.3180	**1.2698**	0.2400	1.700	**3.4200**

Lei-1 [19]	0.0	0.0	18.352			3.1500		
Lei-2	(given)	0.4787	3.5735	**0.5318**		0.5735	**4.74783**	
Lei-3	0.00043	0.4786	2.1442	1.4295	**0.5332**	0.4639	0.6943	**4.7584**

**Table 3:** Calculated constants for the DIN luminescence standard for different grades from A to G, where the luminous intensity increases as the grade number goes from A to G. A double exponential equation was used for interpretation.

Terms	α_1_	α_2_	τ_1_ (min)	τ_2_ (min)
A	0.1587	0.0162	3.7138	34.8572
B	0.2934	0.0314	4.0907	39.0857
C	1.0415	0.1004	3.5680	36.8189
D	1.5453	0.2046	3.7503	33.6278
E	2.5939	0.2570	3.9084	38.4057
F	3.2869	0.3366	3.9448	37.8302
G	4.3611	0.4439	3.8110	34.8920

**Table 4:** Fitting parameters of the experimental decay curves conducted in this study, where the value 4 denotes 0.4 mm and the value 6 denotes 0.6 mm in thickness; the values 0050, 0200, 1000, and 3000 represent the luminous intensity of excitation. A double exponential equation was applied in interpretation.

Name	α_1_	α_2_	τ_1_ (min)	τ_2_ (min)
4-0050	0.5802	**0.2180**	1.044	**12.8277**
4-0200	6.4165	**0.5041**	0.7377	**13.8277**
4-1000	7.0333	**0.7574**	1.0377	**12.734**
4-3000	5.489	**1.0582**	1.6433	**12.414**
6-0050	0.2803	**0.1627**	2.6577	**16.948**
6-0200	1.2105	**0.5252**	2.5777	**15.6677**
6-1000	4.8986	**1.0896**	1.351	**10.961**
6-3000	10.3447	**1.3694**	1.2643	**13.068**
